# The role of human and mosquito behaviour in the efficacy of a house-based intervention

**DOI:** 10.1098/rstb.2019.0815

**Published:** 2020-12-28

**Authors:** Antoine M. G. Barreaux, Welbeck A. Oumbouke, N'Guessan Brou, Innocent Zran Tia, Ludovic P. Ahoua Alou, Dimi Théodore Doudou, Alphonsine A. Koffi, Raphaël N'Guessan, Eleanore D. Sternberg, Matthew B. Thomas

**Affiliations:** 1 Center for Infectious Disease Dynamics and Department of Entomology, Pennsylvania State University, University Park, PA 16802, USA; 2 School of Biological Sciences, University of Bristol, Bristol BS8 1TQ, UK; 3 Institut Pierre Richet/Institut National de Santé Publique (INSP), Bouaké, Cote d'Ivoire; 4 London School of Hygiene and Tropical Medicine, Keppel Street, London WC1E 7HT, UK; 5 Centre de recherche pour le Développement (CRD)/Laboratoire de Santé, Nutrition et Hygiène, Université Alassane Ouattara, Bouaké BP V 18 01, Cote d'Ivoire; 6 Liverpool School of Tropical Medicine, Pembroke Place, Liverpool L3 5QA, UK

**Keywords:** housing improvement, vector control, screening, EaveTubes, mosquito behaviour, human behaviour

## Abstract

Housing improvement such as blocking eaves and screening windows can help in reducing exposure to indoor biting mosquitoes. The impacts of physical barriers could potentially be boosted by the addition of a mechanism that kills mosquitoes as they attempt to enter the house. One example is to combine household screening with EaveTubes, which are insecticide-treated tubes inserted into closed eaves that attract and kill host-searching mosquitoes. The epidemiological impact of screening + EaveTubes is being evaluated in a large cluster randomized trial in Cote d'Ivoire. The study presented here is designed as a complement to this trial to help better understand the functional roles of screening and EaveTubes. We began by evaluating householder behaviour and household condition in the study villages. This work revealed that doors (and to some extent windows) were left open for large parts of the evening and morning, and that even houses modified to make them more ‘mosquito proof’ often had possible entry points for mosquitoes. We next built two realistic experimental houses in a village to enable us to explore how these aspects of behaviour and household quality affected the impact of screening and EaveTubes. We found that screening could have a substantial impact on indoor mosquito densities, even with realistic household condition and behaviour. By contrast, EaveTubes had no significant impact on indoor mosquito density, either as a stand-alone intervention or in combination with screening. However, there was evidence that mosquitoes recruited to the EaveTubes, and the resulting mortality could create a community benefit. These complementary modes of action of screening and EaveTubes support the rationale of combining the technologies to create a ‘Lethal House Lure’.

This article is part of the theme issue ‘Novel control strategies for mosquito-borne diseases’.

## 1. Introduction

Contemporary control of malaria mosquitoes relies heavily on the core technologies of long-lasting insecticidal nets (LLIN) and indoor residual spraying (IRS) of insecticides [1]. However, in spite of their considerable impact [2], it is widely acknowledged that additional tools are required to achieve the control targets set out in the WHO Global Technical Strategy for malaria [1,3]. Housing improvement has been used as a vector control strategy for centuries [4,5] and has received renewed attention in recent times, not only with respect to vector borne diseases but also as a means of improving human health more generally [4,6–9]. There are a range of household modifications that have been shown to reduce mosquito–human contact rate including blocking eaves (the gap between the top of the wall and the roof of the house) and screening doors and windows, with some evidence for impact of these approaches on ultimate disease burden [7,10–12].

One recent variation on the theme of house modification is what the WHO Vector Control Advisory Group (VCAG) (https://apps.who.int/iris/bitstream/handle/10665/274451/WHO-CDS-VCAG-2018.03-eng.pdf) have called a ‘Lethal House Lure’. This approach aims to modify the house in some way so that rather than simply blocking entry with a physical barrier, the house becomes a ‘lure and kill’ device that targets malaria mosquitoes as they host search and attempt to feed indoors at night. One version of a Lethal House Lure combines general house improvement (e.g. blocking eaves, patching holes in walls, fitting screening to windows) with In2Care EaveTubes [13]. The EaveTubes are pieces of 16.5 cm diameter PVC (polyvinyl chloride) pipe fitted into the closed eaves of a house, with typically 8–10 tubes per house. The tubes act something like chimneys to channel human odour cues out of the house. When mosquitoes follow these odour plumes, they enter the tubes and contact an insecticide-treated screen able to kill even insecticide-resistant mosquitoes [14,15]. A range of semi-field studies suggest that screening + EaveTubes can reduce entry of mosquitoes and increase overnight mosquito mortality rate [14,16–19]. For example, adding EaveTubes to WHO-style west African experimental huts [20] reduced mosquito entry by 60% and blood feeding by 65% [16]. Furthermore, experiments conducted in semi-field enclosures showed the overall cumulative mortality from screening + EaveTubes to be around 90% over four nights as mosquitoes attempted to enter huts on successive nights [17], with no evidence for deflection of mosquitoes from huts with screening + EaveTubes to adjacent unmodified huts [16].

The epidemiological impact of screening + EaveTubes is currently being evaluated in a large-scale cluster randomized trial (CRT) in 40 villages in central Côte d'Ivoire. Full details of the study site, the study design and the evaluation protocols are provided in Sternberg *et al*. [21]. In brief, the CRT took place between 2016 and 2019 in the Gbêkê region in central Côte d'Ivoire. This region has year-round malaria transmission, with a peak during the wet season (May–October). The local malaria vector populations are dominated by *Anopheles gambiae* s.l. that are highly resistant to almost all classes of insecticides currently being used for vector control. In the CRT, 20 villages received the screening + EaveTubes treatment, while the other 20 villages acted as the unmodified controls. All villages received LLINs at universal coverage as a standard baseline intervention. Key endpoints were the incidence of clinical malaria cases in cohorts of children in the study villages, together with secondary entomological endpoints including density of mosquitoes indoors and outdoors, and entomological inoculation rate. The research presented in the current paper was designed to complement this CRT by trying to better understand the relative impacts of screening and EaveTubes on indoor mosquito densities at household level. We began by surveying household behaviour and house condition in the study villages in order to determine the likely effectiveness of screening in preventing mosquito entry. This work revealed that many houses had possible entry points for mosquitoes and that, irrespective of house quality, doors and windows were often open through periods of the evening and morning when malaria mosquitoes were likely to be host searching. Based on these data, we next conducted a series of experiments using two experimental houses built within a study village to enable us to simulate a range of conditions and partition the effects of screening, EaveTubes and human behaviour on indoor mosquito densities. The aim of these experiments is to aid the ultimate interpretation of the results from the CRT and guide future development and testing of the Lethal House Lure approach. In addition, they provide general insights into factors affecting the effectiveness of house-based interventions.

## 2. Methods

### (a) Assessment of householder behaviour

Data were collected to assess householder behaviour considering whether doors and windows were open in individual houses, and whether occupants were awake or asleep. The data were collected hourly between 18.00 and 8.00 by the technicians responsible for supervising mosquito sampling in the main CRT. For each hour, the technicians recorded whether the door was open to the house, whether the windows were open and whether household members were still awake, with each category scored as ‘yes’, ‘no’ or ‘don't know’. Data were collected from 1120 randomly selected houses over 70 sampling nights between December 2017 and June 2018 (each month, 4 houses per village across 40 villages), which spanned the start of the dry season to the middle of the rainy season in the second year of the CRT.

### (b) Assessment of house quality

CRT village houses were assessed for quality and potential mosquito entry points. The houses were examined from the outside and the presence of holes or openings in any window screening, windows, the door (including gaps above or below the door), eaves and walls were recorded if they were considered large enough to allow access to mosquitoes (i.e. at least 1 cm diameter or width) and if they fully penetrated the house. Assessments were conducted during September and October 2018 and then again during November and December 2018, with 800 randomly selected houses on each occasion (400 houses from the treatment villages that received screening and EaveTubes and 400 in the control villages that received no household modifications).

### (c) Experimental houses

**Figure 1.**
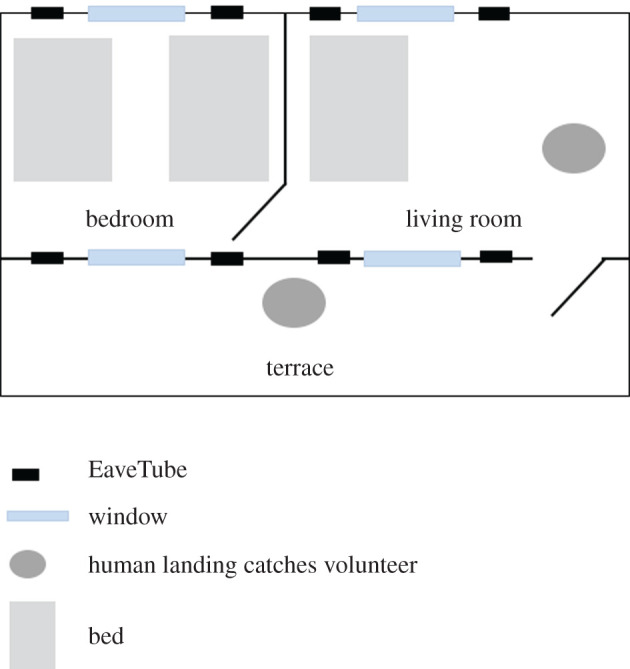
Plan of an experimental house.

**Figure 2.**
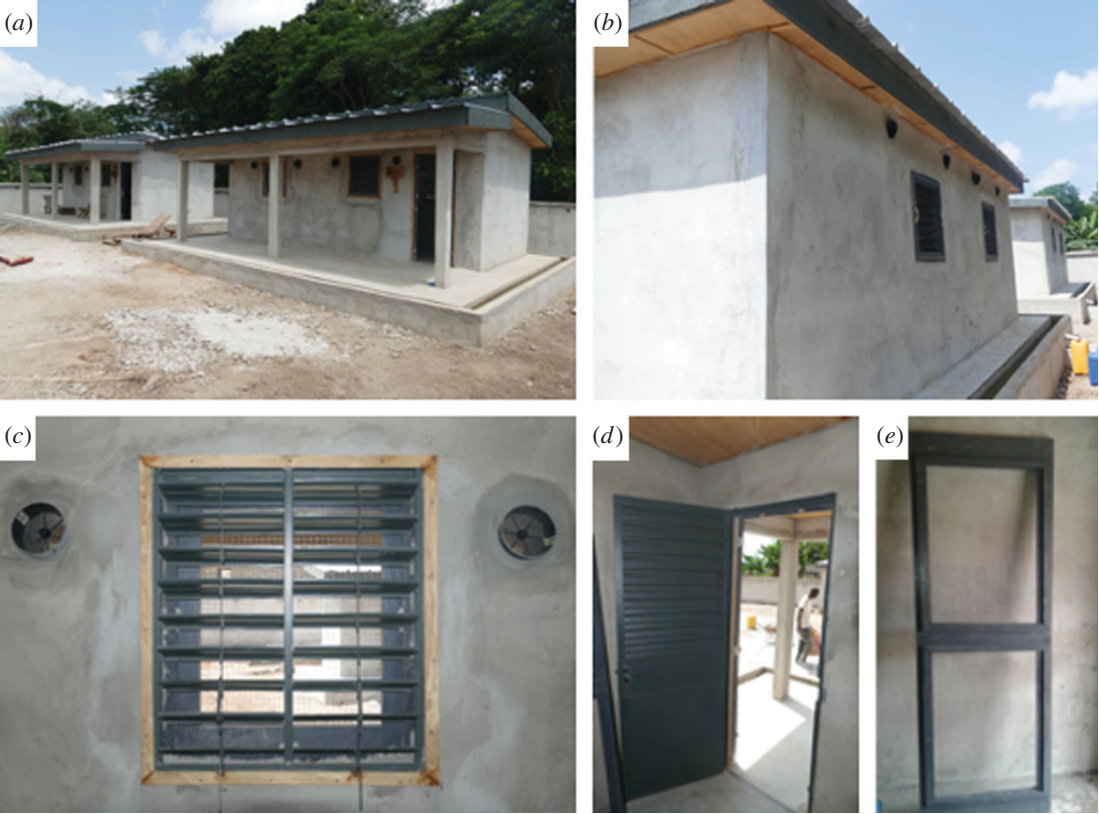
Pictures of the experimental houses. (*a*) The two experimental houses; (*b*) back of a house showing EaveTubes; (*c*) metallic window from the inside; (*d*) metallic front door; (*e*) chicken wire frame to put on the front door, which allowed mosquito entry but prevented entry of reptiles and rats when the doors were open.

Two identical houses were built next to each other in one of the control villages from the CRT (the village of Kologonouan; 7.674768, −5.162976) following a typical house design for the local area. Each house had one bedroom and one living room, and a covered terrace (figure 1). There were two windows in the living room and two in the bedroom (one in the front and one in the back). The houses were constructed of brick and cement, with metal roofs, wooden ceilings, and metal doors and windows with louvres (figure 2). The windows and doors were also equipped with removable mosquito-proof screening. When the screening was removed, it was replaced with panels of chicken wire that allowed natural airflow and unhindered access by mosquitoes but prevented access by reptiles and rodents when the doors and windows were left open. Four holes to accommodate eave tubes were drilled per room (two in the front and two in the back) which is consistent with the density of tubes per house in the CRT.

### (d) Insecticide treatment

In2care EaveTubes comprise plastic inserts containing netting treated with an electrostatic coating (In2Care Insectech^®^, The Netherlands) that are placed within the PVC pipe. The coating provides a positive charge that enables insecticide powders to bind to the netting. Inserts block mosquito entry and contact with the netting leads to transfer of insecticidal particles onto the mosquito body, delivering sufficiently high dose to potentially overcome insecticide resistance [13,15]. For the current study, inserts were treated with a wettable powder formulation of 10% β-cyfluthrin (Tempo 10 ©, Bayer) at a range of 300–500 mg of powder per insert, which is the same as the ‘proof of principle’ treatment used in the CRT in Cote d'Ivoire. Such treated inserts have been shown to kill wild, highly pyrethroid-resistant mosquitoes in a range of laboratory, semi-field and field studies [14,16,17].

### (e) Household occupants and mosquito sampling

Each house had three sleepers, two in the bedroom and one in the living room. These adult volunteers slept from 20.00 to 6.00 and were provided with LLINs (Permanet 2.0). In addition, there were two volunteers responsible for sampling mosquitoes using human landing catches (HLC), one in the living room and one outside on the terrace. HLCs were conducted from 18.00 to 8.00. Halfway through each sample night, the HLC volunteers were replaced by a second pair of volunteers. Capturers were seated with bare legs from the knees down and collected any mosquitoes landing on their legs using haemolysis glass tubes and a flashlight. There were additional supervisors for each house to ensure the HLC volunteers were awake and to open and close doors and windows as appropriate.

Mosquitoes that were collected were brought back for identification to the species level using a binocular microscope (×40) at the Institut Pierre Richet (IPR) research centre in Bouake, Côte d'Ivoire. The data presented in the current study focus just on *An. gambiae* (s.l.) mosquitoes that are the primary malaria vector in the region and were by far the numerically dominant species.

### (f) Experimental house studies

#### (i) Experimental house study (1): impact of human behaviour and screening/EaveTubes on mosquito entry

This experiment aimed to evaluate how householder behaviour influenced the impact of screening + EaveTubes on indoor density of mosquitoes and also whether EaveTubes alone could reduce mosquito entry rate. Accordingly, mosquito captures were compared between a typical house without screening or EaveTubes (representative of the control arm in the CRT), a house with screening + EaveTubes (representative of the treatment arm in the CRT) and a house with EaveTubes alone. Houses without EaveTubes had ‘closed’ eaves, meaning tubes closed with pieces of tarpaulin to block mosquito entry and prevent airflow. In addition, we varied householder behaviour so that either doors and windows were shut throughout the sampling period (‘modified’ behaviour), or they were open through part of the evening and morning in line with the ‘typical’ behaviour observed in the study villages (table 1). Specifically, for the ‘typical’ behaviour, windows were open from 18.00 to 20.00, closed from 20.00 to 6.00, and open again from 6.00 to 8.00, while the front door was open from 18.00 until midnight, closed from midnight until 5.00 and then open from 5.00 to 8.00. During daytime non-test periods (i.e. 8.00–18.00), doors and windows were kept closed to prevent incidental mosquito entry.

**Table 1.** The different house typologies used in experimental house studies. ‘Typical’ householder behaviour means that doors and windows are open in the evening and morning whereas ‘modified’ means that everything is closed all night long. ‘Closed’ eaves mean tubes closed with pieces of tarpaulin to block mosquito entry and prevent airflow. ‘Open’ eaves mean that the plastic tubes of the EaveTubes are left open with no inserts in place to block mosquito entry. By standard house, we mean a typical house design for the villages around the city of Bouake in Cote d'Ivoire. The houses were made of brick and cement, with metal roofs, wooden ceilings, and metal doors and windows with louvres (figure 2) as observed in the area. Each house had one bedroom and one living room, and a covered terrace (figure 1). There were two windows in the living room and two in the bedroom (one in the front and one in the back).

house typologies	standard house	screening + EaveTubes ‘typical’	screening + EaveTubes ‘modified’	EaveTubes	screening	screening + open EaveTubes
householder behaviour	typical	typical	modified	typical	typical	typical
eaves	closed	EaveTubes	EaveTubes	EaveTubes	closed	open
windows	open window slits	screening	screening	open window slits	screening	screening
Experiment 1	✓	✓	✓	✓		
Experiment 2	✓	✓		✓	✓	
Experiment 3	✓	✓		✓	✓	✓

The different house typologies were assessed two-by-two, with treatments rotated over both experimental houses and with two teams of household volunteers (capturers and sleepers) also rotated. There were 48 sample nights representing 24 times for each house type, 12 times for each house (or team of volunteers) and 6 times for each combination of house and volunteer team.

#### (ii) Experimental house study (2): determining the relative contribution of screening and EaveTubes in houses of good condition

This experiment aimed to determine more explicitly the relative roles of screening and EaveTubes in reducing indoor mosquito densities. We compared a standard control house with no screening or EaveTubes with a house with screening + EaveTubes, a house with EaveTubes alone and a house with screening alone (table 1). Houses without EaveTubes had ‘closed’ eaves, meaning tubes closed with pieces of tarpaulin to block mosquito entry and prevent airflow. On this occasion, all houses had ‘typical’ human behaviour (i.e. doors and windows open as per the behaviour observed in the study villages). Again, house typologies were assessed two-by-two and rotated over both houses and the two teams of volunteers. There were 48 total sample nights representing 24 times for each house type, 12 times for each house (or team of volunteers) and 6 times for each combination of house and volunteer team.

#### (iii) Experimental house study (3): determining the relative contribution of screening and EaveTubes in houses with condition more representative of typical village houses

Although the two experimental houses were designed to be representative of the local housing, because they were new and well built, they were still somewhat atypical of many village houses that tend to have damage to the walls or eaves, holes in the screening, and gaps around the doors (see Results and table 2). Accordingly, the two experimental houses were modified by adding a 1 cm gap at the top and bottom of the door, and creating 4 × 4 cm squares holes in each of the four corners of the window screening (analogous to how bed nets are damaged in standard WHO tests [22]). The eaves were not modified as there was no easy way to mimic the varied openings observed in some of the village houses. However, removing the inserts from the eave tubes provided a mechanism to allow mosquitoes to access the houses via the eaves.

**Table 2.** Results from the house quality survey conducted in a random subset of houses from 40 villages in central Cote d'Ivoire in September and November 2018. The number of houses inspected for condition in the control villages and the screening + EaveTubes villages is given, together with the mean (and standard error, s.e.) percentage of houses with damage to either window screening, the front door, eaves or walls. Damage is defined as at least one hole of sufficient size (greater than 1 cm diameter or width) that could potentially allow mosquito access.

		September 2018		November 2018	
		control	screening + EaveTubes	control	screening + EaveTubes
houses (number)		404	408	401	399
holes in screening (%)	mean (s.e.)	—	48.0 (2.56)	—	6.0 (1.19)
gaps in front door (%)	mean (s.e.)	87.6 (1.64)	81.3 (1.93)	92.5 (1.32)	73.7 (2.21)
openings in eaves (%)	mean (s.e.)	35.5 (1.50)	41.5 (1.55)	35.7 (2.39)	14.5 (1.77)
holes in walls (%)	mean (s.e)	14.2 (1.00)	18.4 (1.11)	20.2 (2.01)	11.3 (1.59)

With these modified houses, we again examined the relative contributions of screening and EaveTubes comparing a typical control house with no screening or EaveTubes, a house with screening + EaveTubes, a house with EaveTubes alone and a house with screening alone (table 1). Houses without EaveTubes had ‘closed’ eaves, meaning tubes closed with pieces of tarpaulin to block mosquito entry and prevent airflow. In addition, we also included a fifth treatment comprising a house with screening but this time open eaves (i.e. the inserts removed from the tubes) to examine whether screening has any impact when there are gaps in the eaves and also to provide a measure of the number of mosquitoes that recruit to the EaveTubes but are not reflected in the indoor catches because they are prevented from entering by inserts. All houses had ‘typical’ human behaviour. Again, house typologies were assessed two-by-two and rotated over both experimental houses. There were 40 nights of capture, giving 16 replicates for each house type and 8 for each house.

### (g) Sample size calculation

The number of replicate nights for each experimental house study was determined in the first instance based on practical constraints of time and personnel. However, replication was checked retrospectively based on the empirical data using the ‘pwr’ package v. 1.2-2 in R v. 3.6.2 [23]. The number of sample nights was above the number required to demonstrate 5% significance with greater than 80% power.

### (h) Analysis

#### (i) Number of mosquitoes captured per house per night

Analyses of variance incorporating random effects (night of capture, house and volunteer team) were performed to assess differences in mosquito captured inside and outside per house and per night between house types. House typology was considered the single fixed effect or subdivided in up to three fixed effects: window screening (present or not), EaveTubes (insecticide-treated insert, closed eaves or open eaves) and human behaviour (‘typical’ behaviour, or doors and windows always closed from 18.00 to 8.00). Resulting linear mixed models were obtained in the software R v. 3.6.2, using the lme4 package, v. 1.1-21, and the ‘lmer’ function. The number of *An. gambiae* captured per house and per night was log transformed when needed so that the residuals of the models followed a normal distribution.

The likelihood ratio test (LRT) was used to fit and simplify models for random effects. A random effect was removed if a model with a given random effect was not significantly different from the same model without this random effect (*p*-value > 0.05). Model comparison was done with the ‘anova’ function in the package lme4 and the maximum-likelihood method (ML) [24–27]. The house type, the impact of screening, EaveTubes and human behaviour (fixed effects) in the fitted linear mixed models were analysed using the restricted maximum-likelihood (REML) approach (packages ‘lme4’ v. 1.1-21 and ‘lmerTest’ v. 3.1-1) and the Kenward–Roger approximation [27–29]. Fixed effects with *p*-values > 0.05 were considered not significant. After making sure that there were significant differences between house typologies for mosquito capture, a *post hoc* test was used to compare house typologies two-by-two using the function ‘difflsmeans’ in the ‘lmerTest’ package.

#### (ii) Experimental house study (1): impact of human behaviour and screening/EaveTubes on mosquito entry

We analysed the log-transformed number of *An. gambiae* captured per house and per night, with screening, EaveTubes and human behaviour as fixed effects and the night of capture, the house and the team of volunteers as random effects. We also analysed the number of *An. gambiae* captured per house and per night regarding the house typology as a fixed effect and the night of capture, the house and the team of volunteers as random effects. A *post hoc* test was then performed for pairwise comparisons between house typologies.

#### (iii) Experimental house study (2): determining the relative contribution of screening and EaveTubes in houses of good condition

We analysed the log-transformed number of *An. gambiae* captured per house and per night, with screening, EaveTubes and their interaction as fixed effects and the night of capture, the house and the team of volunteers as random effects. We also analysed the number of *An. gambiae* captured per house and per night regarding the house typology as a fixed effect and the night of capture, the house and the team of volunteers as random effects. A *post hoc* test was then performed for pairwise comparisons between house typologies.

#### (iv) Experimental house study (3): determining the relative contribution of screening and EaveTubes in houses with condition more representative of typical village houses

Considering all house typologies except the one with open eaves, we analysed the number of *An. gambiae* captured per house and per night, with screening, EaveTubes and their interaction as fixed effects and the night of capture and the house (the effect of the volunteer team is part of the house effect here) as random effects.

Considering all five house typologies, we then analysed the number of *An. gambiae* captured per house and per night, with the house typology as a fixed effect and the night of capture and the house (the effect of the volunteer team is part of the house effect here) as random effects. A *post hoc* test was then performed for pairwise comparisons between house typologies.

## 3. Results

### (a) Assessment of householder behaviour

**Figure 3.**
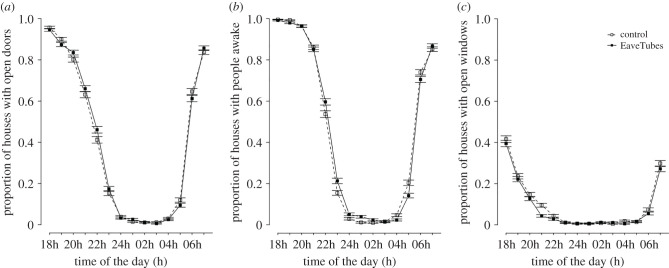
Proportion of houses with open doors (*a*), people awake (*b*) or open windows (*c*). It is given regarding the CRT treatment arm, control or EaveTubes+screening and it was assessed between December 2017 and June 2018.

The results of the observational survey of householder behaviour are summarized in figure 3. There was virtually no difference in behaviour between treatment and control villages from the CRT. The majority of houses had their doors open at 18.00 and this then showed a gradual decline until 12.00, when nearly all houses had their doors shut, paralleling when the household members were asleep. Doors then remained closed until 4.00–5.00 when the household members started to wake up and by 7.00, most houses had their doors open again. Opening and closing of windows followed a similar pattern, although the windows tended to be closed up slightly earlier in the evening and fewer windows tended to be open in total at household level.

### (b) Assessment of house quality

The survey revealed that many houses had some sort of damage that could potentially allow mosquito access (table 2). The majority of doors either had holes or gaps and this was largely consistent between control and treatment villages. The percentage of houses with damaged walls was much lower but was again consistent between treated and control villages. Around 35% of control houses had gaps in part of the eaves. For houses in the treatment arm, the gaps in the eaves were marginally higher (41.5%) than controls during the September survey but lower in the November survey (14.5%). This reduction between September 2018 and November 2018 likely reflects the fact that the implementation team in the CRT visited the houses in the treatment villages 2–3 times per year to conduct basic repairs to maintain the ‘mosquito proofing’, and one of these repair rounds occurred during this period. The effect of repairs can also be seen in the window screening, whereby 48% of the houses surveyed in the treatment villages in September had some sort of damage, whereas this was only 6% for houses surveyed in November (note, however, that houses were randomly selected in the villages so there could also be a sampling effect here as the same houses were not necessarily inspected on both occasions).

### (c) Experimental house study (1): impact of human behaviour and screening/EaveTubes on mosquito entry

**Figure 4.**
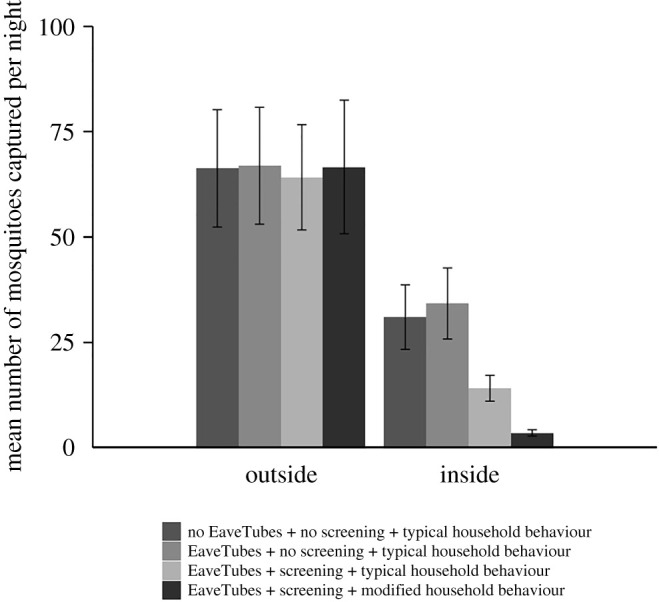
Mean (±s.e.) number of *An. gambiae* mosquitoes captured outside or inside experimental houses per night, depending on house type (Experiment 1). There were three house typologies: a standard village style house with closed eaves but no EaveTubes or screening; houses fitted with EaveTubes alone and no screening; houses fitted with screening + EaveTubes. In addition, householder behaviour was managed to either reflect typical behaviour in which doors and windows were open for part of the evening and morning (see Methods and figure 3), or behaviour was modified so that doors and windows were kept closed from 18.00 to 8.00. Mosquitoes were collected by HLC from 18.00 to 8.00. The means represent 48 nights of capture with individual treatments replicated 24 times.

There was no effect of screening (*F*_1,47_ = 0.16, *p* = 0.700), EaveTubes (*F*_1,47_ = 0.34, *p* = 0.565) or human behaviour (*F*_1,47_ = 1.23, *p* = 0.27) on the number of *An. gambiae* mosquitoes captured outside the houses each night (figure 4). On average (mean ± s.e.), 66.9 ± 13.89 mosquitoes were captured per night outside a house with just EaveTubes; 66.6 ± 15.89 mosquitoes outside a house with screening + EaveTubes and everything closed; 66.3 ± 13.95 mosquitoes outside a standard control house; and 64.1 ± 12.51 mosquitoes outside a house with screening + EaveTubes and typical behaviour where windows and doors were open during evening and morning. There was no effect of the house or the team of volunteers on mosquito numbers (*p* > 0.05), but there were differences between capture nights (*χ*^2^ = 94.98, d.f. = 1, *p* < 0.001).

**Figure 5.**
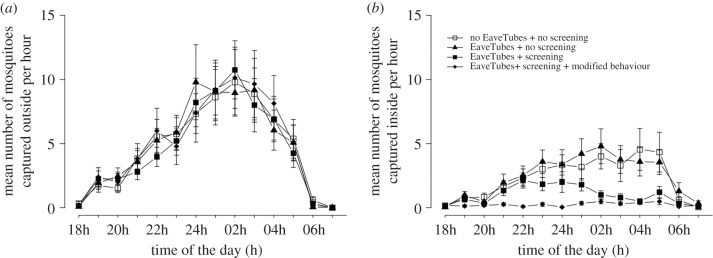
Mean (±s.e.) number of *An. gambiae* mosquitoes captured per hour outside or inside experimental houses, depending on house type (Experiment 1). There were three house typologies: a standard village style house with closed eaves but no EaveTubes or screening; houses fitted with EaveTubes alone and no screening; houses fitted with screening + EaveTubes. In addition, householder behaviour was managed to either reflect typical behaviour in which doors and windows were open for part of the evening and morning (see Methods and figure 3), or behaviour was modified so that doors and windows were kept closed from 18.00 to 8.00. Mosquitoes were collected by HLC from 18.00 to 8.00. The means represent 48 nights of capture with individual treatments replicated 24 times.

Hourly mosquito captures showed that *An. gambiae* began host searching at around 18.00–19.00, peaked at 12.00–2.00 and then declined to negligible levels by 6.00–7.00 (figure 5; note these patterns were qualitatively consistent across all experiments regardless of whether captures were indoors or outdoors, and so we present one representative dataset to illustrate this behaviour).

In contrast with the outdoor catches, there were significant differences between the house typologies with respect to the numbers of *An. gambiae* mosquitoes captured indoors (*F*_3,54_ = 18.20, *p* < 0.001) (figure 4). EaveTubes did not influence indoor mosquito numbers (*F*_1,54_ = 0.16, *p* = 0.69). Houses with EaveTubes alone had marginally higher indoor mosquito numbers compared to a standard control house, but the difference was non-significant (34.2 ± 8.44 compared with 31.0 ± 7.67 mosquitoes per house, respectively; *t*-value = 0.40, d.f. = 54, *p* = 0.693).

Adding screening significantly decreased entry of *An. gambiae* (*F*_1,54_ = 9.53, *p* = 0.003). When considering only houses with typical human behaviour (i.e. doors and windows open for parts of the evening and morning), there was a 55% decrease in mosquito entry for a house with screening + EaveTubes compared to a standard control house (14.0 ± 3.08 and 31.0 ± 7.67 mosquitoes per night, respectively; *t*-value = 3.48, d.f. = 54, *p* = 0.001) (figure 4).

When householder behaviour was modified to keep doors and windows closed, household entry was reduced further (*F*_1,54_ = 8.86, *p* = 0.004), with an 89% reduction in mosquito entry compared to a standard house with typical behaviour (3.4 ± 0.75 and 31.0 ± 7.67, respectively; *t*-value = 6.46, d.f. = 54, *p* < 0.001), and a 76% reduction compared to a house with screening and EaveTubes with typical behaviour (3.4 ± 0.75 and 14.0 ± 3.08, respectively; *t*-value = 2.98, d.f. = 54, *p* = 0.004) (figure 4).

These treatment effects are further illustrated in the hourly indoor catches, which show no differences between a standard control house or a house with EaveTubes alone, a very low level of recruitment in a house with screening + EaveTubes and modified behaviour, and an intermediate pattern with screening + EaveTubes and typical behaviour where mosquitoes can be seen entering the house when the doors and windows were open, but then showing a decline when the house was closed up (figure 5).

There was no effect of the house or the team of volunteers on indoor mosquito captures (*p* > 0.05), but there were differences between nights (*χ*^2^ = 28.56, d.f. = 1, *p* < 0.001).

### (d) Experimental house study (2): determining the relative contribution of screening and EaveTubes in houses of good condition

There was no effect of screening (*F*_1,48_ = 2.07, *p* = 0.156), EaveTubes (*F*_1,48_ = 0.0033, *p* = 0.954) or their interaction (*F*_1,48_ = 1.19, *p* = 0.28) on the number of *An. gambiae* captured outside the houses each night. On average, 46.0 ± 5.65 mosquitoes were recaptured per night outside houses fitted with just EaveTubes, 66.1 ± 7.58 in houses with just screening, 54.9 ± 7.15 in houses fitted with screening + EaveTubes and 55.8 ± 6.74 in control houses. There were no significant effects of the house or the volunteers (*p* > 0.05), but there were some significant differences between nights of capture (*χ*^2^ = 65.23, d.f. = 1, *p* < 0.001).

There were significant differences between the house typologies in indoor mosquito density (*F*_3,58_ = 8.16, *p* < 0.001). As in the previous experiment, EaveTubes alone did not significantly reduce the number of *An. gambiae* captured indoors (*F*_1,58_ = 1.75 *p* = 0.19); there was a marginal decrease compared with the standard control house (31.8 ± 4.54 compared with 41.6 ± 5.78, respectively), but this was not significant (*t*-value = 1.32, d.f. = 58, *p* = 0.191).

**Figure 6.**
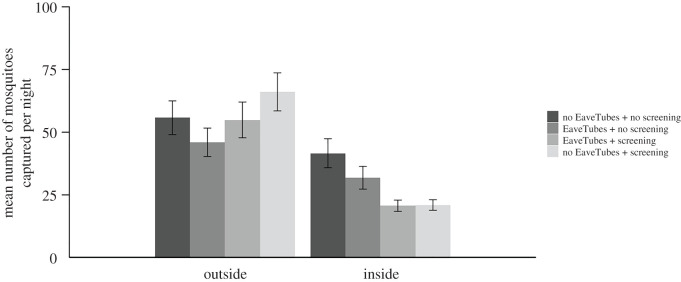
Mean (±s.e.) number of *An. gambiae* mosquitoes captured outside or inside experimental houses per night, depending on house type (Experiment 2). There were four house typologies: a standard village style house with closed eaves but with no EaveTubes or screening; houses fitted with EaveTubes alone and no screening; houses fitted with no EaveTubes (closed eaves) and screening alone; or houses fitted with screening + EaveTubes. Householder behaviour was managed to reflect typical behaviour in which doors and windows were open for part of the evening and morning (see Methods and figure 3). Mosquitoes were collected by HLC from 18.00 to 8.00. The means represent 48 nights of capture with individual treatments replicated 24 times.

On the other hand, screening alone did decrease mosquito entry (*F*_1,58_ = 15.78, *p* < 0.001). The mean indoor mosquito density was about 50% lower in either a screened house (20.9 ± 2.09; *t*-value = 4.10, d.f. = 58, *p* < 0.001) or a house with screening + EaveTubes (20.6 ± 2.23; *t*-value = 3.97, d.f. = 58, *p* < 0.001) compared to a standard control house (41.6 ± 5.78), and there was no apparent benefit of adding EaveTubes to screening in terms of mosquito entry (*F*_1,58_ = 1.06 *p* = 0.31) (figure 6).

There were no effects of the house or the volunteers (*p* > 0.05), but there were some significant differences between nights of capture (*χ*^2^ = 18.90, d.f. = 1, *p* < 0.001).

### (e) Experimental house study (3): determining the relative contribution of screening and EaveTubes in houses with condition more representative of typical village houses

**Figure 7.**
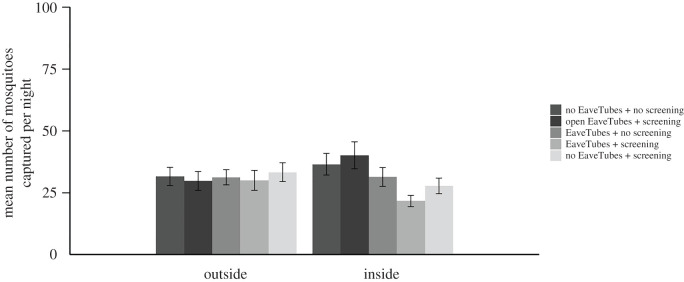
Mean (±s.e.) number of *An. gambiae* mosquitoes captured outside or inside experimental houses per night, depending on house type (Experiment 3). There were five house typologies: a standard village style house with closed eaves but with no EaveTubes or screening; houses fitted with EaveTubes alone and no screening; houses fitted with no EaveTubes (closed eaves) and screening alone; houses fitted with screening + EaveTubes; or houses fitted with screening but with the EaveTubes inserts removed so that the eaves were open. Householder behaviour was managed to reflect typical behaviour in which doors and windows were open for part of the evening and morning (see Methods and figure 3). Furthermore, the window screening was deliberately damaged (four 4 × 4 cm holes added per window) and the doors modified to create a 1 cm gap above and below the door to make the house condition more representative of a typical village house. Mosquitoes were collected by HLC from 18.00 to 8.00. The means represent 40 nights of capture with individual treatments replicated 16 times.

Again, there was no effect of screening or EaveTubes on outside capture (*p* > 0.05). There were some significant differences between nights of capture (*χ*^2^ = 14.78, d.f. = 1, *p* < 0.001) but not between houses (*p* > 0.05) (figure 7).

Considering first the four basic house typologies, we found significant differences (*F*_3,40_ = 3.63, *p* = 0.021) between house typologies that broadly followed similar patterns to previous experiments. Adding EaveTubes alone did not decrease mosquito entry (*F*_1,41_ = 1.26, *p* = 0.268); indoor densities were lower in houses with just EaveTubes compared with standard control houses (mean densities of 31.4 ± 3.79 and 36.5 ± 4.36 mosquitoes per house per night, respectively) but the effect was not significant (*t*-value = 0.72, d.f. = 40, *p* = 0.478) (figure 7).

Adding screening significantly decreased *An. gambiae* mosquito entry (*F*_1,41_ = 9.89, *p* = 0.003) though the effect size was smaller than in previous experiments. Indoor mosquito density was reduced by 24% in houses with screening alone compared with control houses (27.8 ± 3.16 and 36.5 ± 4.36 mosquitoes per house per night, respectively; *t*-value = 2.11, d.f. = 40, *p* = 0.041). The combination of screening + EaveTubes reduced densities further (21.6 ± 2.25 mosquitoes per house per night compared with 36.5 ± 4.36 in the control; *t*-value = 2.99, d.f. = 40, *p* = 0.005), but this was not significantly different from screening alone (*t*-value = 0.88, d.f. = 40, *p* = 0.382) (figure 7).

There were some significant differences between nights of capture (*χ*^2^ = 14.78, d.f. = 1, *p* < 0.001) and between the two houses (*χ*^2^ = 7.90, d.f. = 1, *p* = 0.005).

Considering the fifth experimental house type (*F*_4,47_ = 5.03, *p* = 0.002), opening the eaves by removing the inserts from the PVC tube of the EaveTubes led to the highest indoor density (40.1 ± 5.43 *An. gambiae* per house per night), which represents an increase in mosquito entry of 85% compared to a house with screening and intact EaveTubes (*t*-value = 4.01, d.f. = 46, *p* < 0.001), and 45% compared to a house with screening and closed eaves (*t*-value = 3.21, d.f. = 47, *p* = 0.002). However, the house with screening and open eaves did not have significantly different mosquito densities to either the standard control house or the house with just EaveTubes and no screening (*p* > 0.05).

## 4. Discussion

This study had two basic aims. First was to help better understand the possible impacts of household screening and EaveTubes on mosquito numbers at household level. This work was done to aid us in the ultimate interpretation of the results of a CRT evaluating this combination of technologies as a ‘Lethal House Lure’ at village scale in Côte d'Ivoire [21]. Second was to provide more general insight into the effectiveness of screening and efforts to make a house more ‘mosquito proof’, and how this might be affected by householder behaviour.

The ‘Lethal House Lure’ approach being tested in the CRT combines efforts to make the house more mosquito proof (filling gaps in eaves and walls where they existed, patching holes in doors and adding screening to windows) with insecticide-treated EaveTubes. The combination of technologies makes intuitive sense as general mosquito proofing ought to reduce house entry (as borne out by previous studies, e.g. [4,7,9,30]) and adding an insecticide should elevate impact beyond a simple physical barrier, potentially enhancing impact at household level [16,17] and providing benefits to the wider community [31]. Nonetheless, interest remains as to the relative contribution of the individual component parts since this could affect the cost-effectiveness of the approach. If, for example, screening has negligible impact then this element could in principle be dropped in favour of using EaveTubes alone, which would reduce the cost of the overall intervention.

The three experimental house studies we conducted were designed to address a series of complementary questions regarding the functioning of screening and EaveTubes at household level. The first experiment focused on the effect of householder behaviour, which was motivated by the results of the field survey showing that householders tend to leave doors open in the evening until the last person goes to bed, and then open the doors when the occupants begin to wake up in the morning. The survey also showed that windows tended to be open for part of the evening, although generally less so than the doors. In principle, open doors and windows could render screening ineffective. We also used this experiment to begin to explore whether EaveTubes alone impacted the number of mosquitoes entering a house at night. This work was motivated by results from earlier experimental hut studies that suggested EaveTubes could reduce indoor densities because the eaves are the preferred entry for mosquitoes [32] and so, if mosquitoes initially recruit to the EaveTubes and are killed (or behaviourally disrupted) by the insecticide, they no longer continue to search around the house for other entry points [16,17]. However, there are differences in the size and general design of experimental huts compared with more realistic houses, so whether these results were transferable to village settings was unclear. Our current results indicate that EaveTubes alone had no impact on reducing mosquito entry rates and it was only the addition of screening that led to reductions in indoor densities. Perhaps not surprisingly, these reductions were most marked when doors and windows were kept closed. However, even when doors and windows were open in line with typical householder behaviour, the screening + EaveTubes treatment still led to significant reductions in mosquitoes indoors relative a standard control house or a house with EaveTubes alone.

The second experimental house study focused more explicitly on the relative contributions of screening versus EaveTubes under conditions of typical householder behaviour. Again, this experiment suggested that EaveTubes alone had no significant impact on indoor mosquito densities in the absence of screening. However, screening alone almost halved indoor mosquito densities even though doors and windows were not shut up throughout the night. Furthermore, there was no difference between screening and screening + EaveTubes, further suggesting a negligible role of EaveTubes at the household level.

The final experimental house study tested EaveTubes versus screening once again, but this time considered more typical house condition as well as typical household behaviour. The survey of house condition from the CRT villages indicated that many houses had damage to the walls, doors, windows, etc., that could potentially allow mosquito access, and this was true even for houses from the treatment arm, especially prior to the repair rounds that were conducted three times a year and were designed to maintain the integrity of the intervention in the study villages. This situation contrasts with our newly built experimental houses where the doors and windows fitted well, and walls and eaves were in good repair. When we deliberately damaged the window screening and added poorly fitting doors we found that the houses were less mosquito proof and there was a suggestion from the data that EaveTubes now played a marginal role in helping to reduce house entry; there was a reduction in indoor density with EaveTubes alone compared to the control house and a greater reduction in EaveTubes + screening compared with screening alone, although these trends were not significant. Removing the insecticide-treated inserts from the EaveTubes reduced any effectiveness of the screening, indicating that if eaves have openings then screening of doors and windows likely has little impact. The increased numbers of mosquitoes indoors when the inserts were removed also provides a measure of the number of mosquitoes that recruit to the EaveTubes but cannot enter when the tubes are intact. We found almost double the number of *An. gambiae* per house per night in houses with screening and open EaveTubes compared with houses with screening and intact EaveTubes. This result confirms the importance of eaves for mosquito entry in real houses, similar to equivalent studies conducted previously in experimental huts [16], and highlights the key potential benefit of EaveTubes; although they do not necessarily contribute to household-level protection, if mosquitoes entering EaveTubes die from contact with insecticide-treated inserts then EaveTubes could contribute to community-wide protection [14–17,31]. This effect is predicted from models [31] and is analogous to the mass action effect of IRS, which provides little benefit at individual household level but does benefit the community when coverage levels are sufficiently high to reduce overall mosquito density and longevity. Such effects cannot be readily detected in household-level experiments such as those conducted here, which is part of the motivation for conducting the village-level CRT [21].

One further area where the effects of EaveTubes could have been underestimated in the current study is that we did not quantify mortality or subsequent fitness of mosquitoes collected indoors. It is possible that some of these mosquitoes could have first visited EaveTubes while searching around the house, before subsequently entering the house via other routes. Studies using experimental huts and field enclosures suggest that transient exposure to an EaveTube can lead to sub- or pre-lethal impacts on traits such as longevity and biting rate [16,17]. Both the lethal and sub-lethal effects of insecticide exposure contribute to the possible impact of EaveTubes and are not necessarily captured at household level.

## 5. Conclusion

Our study confirms that screening can reduce the household entry of malaria mosquitoes, leading to reduced exposure indoors. The level of protection depends on the quality of screening and human behaviour. If the screening is good with no gaps in the eaves or around the doors, and the household occupants close the doors and windows from the evening through to morning, indoor densities can be reduced to very low levels. Encouragingly, even if the house condition is less good and the doors and windows are left open for part of the night, as appears typical of many village houses, there is still a reduction in indoor densities unless, for instance, the eaves have large openings (in our case open eave tubes). These results suggest a concept of ‘mosquito proofing’ analogous to ‘water proofing’. If a house is made of impermeable materials with no holes or gaps, then there will be little water penetration when it rains. If the house has gaps or some damage to the roof, it will tend to leak but is still better than nothing. If these holes are extensive and the doors and windows are left open, then it will likely be just as wet inside as out! The human dimension is important to emphasize here since even the best technology can be undermined if end-user behaviour is not understood. Furthermore, mosquitoes were clearly host searching during the evening and the early morning when householders were not necessarily indoors or asleep. This behaviour provides opportunities for transmission that are potentially unaffected by simple household modifications, or the personal protection effects of LLINs.

In contrast with screening, we found little evidence that EaveTubes reduced mosquito entry rate. This result contradicts some earlier work examining EaveTubes in experimental huts [16], which cautions against extrapolating from very abstracted experimental set-ups to more realistic conditions. Nonetheless, significant numbers of mosquitoes appear to recruit to the EaveTubes and, in doing so, will be exposed to insecticide. While this effect might not contribute very much to household-level protection, reduced mosquito density and/or longevity could contribute to reduced transmission risk at community level, including during the evening periods before people go to sleep and potentially have the house open. These complementary modes of action of screening and EaveTubes support the rationale of combining the technologies to create a ‘Lethal House Lure’.

## Supplementary Material

Supplementary.data.1.

## Supplementary Material

Supplementary.data.2.

## Supplementary Material

Supplementary.data.3.

## Supplementary Material

Supplementary.data.4.

## Supplementary Material

Supplementary.data.5.

## Supplementary Material

Supplementary.data.6.

## Supplementary Material

Supplementary.data.7.

## Data accessibility

The datasets supporting this article have been uploaded as part of the electronic supplementary material.
